# The correlation between lipoprotein(a) and coronary atherosclerotic lesion is stronger than LDL-C, when LDL-C is less than 104 mg/dL

**DOI:** 10.1186/s12872-021-01861-6

**Published:** 2021-01-19

**Authors:** Chuang Li, Qiwen Chen, Mei Zhang, Yin Liu, Yushun Chu, Fanpeng Meng, Jianyu Wang, Jie Tang, Jian Luo, Xiulong Niu, Maoti Wei

**Affiliations:** 1 Faculty of Graduate, The Logistic University of Chinese People’s Armed Police Force, Tianjin, China; 2 Department of Thoracic and Cardiovascular Surgery, Special Medical Center of Chinese People’s Armed Police Force, 220 Chenglin Road, Tianjin, 300162 China; 3 Department of Cardiology, Chest Disease Hospital of Tianjin City, 261 Taierzhuang South Road, Tianjin, 300162 China; 4 Department of Cardiology, Chest Disease Hospital of Tianjin City, 261 Taierzhuang South Road, Tianjin, 300162 China

**Keywords:** Lipoprotein(a), Low-density lipoprotein cholesterol, Acute coronary syndrome, Gensini score

## Abstract

**Background:**

Lp(a) and LDL-C are both risk factors of atherosclerotic cardiovascular disease (ASCVD). But there was a contradiction point in LDL-C and Lp(a) control. The appropriate level of LDL-C and Lp(a) in the prevention of ASCVD is still pending.

**Objective:**

To investigate the correlation of Lp(a) and coronary atherosclerotic lesion, and find out the balance point in LDL-C and Lp(a) control.

**Method:**

3449 patients were divided to coronary atherosclerotic heart disease (CAHD) Group and Non-CAHD Group based on the result of coronary angiography. The clinical characteristics were compared, and Logistic regressions were applied to find the CAHD risk factors in total, High-LDL-C Group (LDL-C ≥ 100 mg/dL) and Low-LDL-C Group (LDL-C < 100 mg/dL) patients. Spearman correlation analysis of Lp(a), LDL-C and Gensini Score was performed in patients with different LDL-C concentration.

**Results:**

Except male and diabetes, the traditional CAHD risk factors were well matched between two groups. But triglyceride, LDL-C and Lp(a) were higher, HDL-C and Apo-A1 were lower in CAHD group (2771). In the Logistic regression analysis, diabetes, LDL-C and Lp(a) are risk factors of CAHD in all patients, while in High-LDL-C Group, they were age, LDL-C, non-HDL-C and ApoB, in Low-LDL-C Group, they were age, Lp(a) and ApoB. Lp(a) correlated with Gensini with coefficient r = 0.41 in all patients, 0.67 in Low-LDL-C Group and 0.32 in High-LDL-C Group. The coefficient r for Lp(a) and Gensini decreased, while the r for LDL-C and Gensini increased with LDL-C concentration increasing. The two fitted lines of rs crossed at LDL-C = 2.7 mmol/L (104 mg/dL).

**Conclusion:**

Lp(a) was the risk factor of CAHD in patients with LDL-C < 100 mg/dL. The correlation between Lp(a) and Gensini was influenced by LDL-C concentration, and the correlation was stronger than LDL-C when LDL-C < 104 mg/dl.

## Background

Atherosclerotic cardiovascular disease (ASCVD) is the leading cause of death and low-density lipoprotein cholesterol (LDL-C) is the well-established risk factor of ASCVD. The epidemiological and Mendelian randomization studies [1, 2] had shown that serum LDL-C level was correlated to ASCVD risk and had significant impact on the clinical outcomes. LDL-C lowering therapies had been proved to reduce ASCVD risk regardless of the patients’ background [3–6]. But the data of 13,167 patients from JUPITER [7], LIPID [8] and AIM-HIGH [9] showed 61% more risk of major adverse cardiovascular event (MACE) in patients with higher lipoprotein (a) (Lp(a)) level than those with similar LDL-C level, but lower Lp(a). That indicated Lp(a) may be another ASCVD risk factor needed to be taken seriously. The relation between Lp(a) and ASCVD has been confirmed by at least 3 meta-analyses [10–12] and recently published result from ODYSSEY Outcomes [13]. The analysis of the Copenhagen City Heart Study and Copenhagen General Population Study showed a higher cardiovascular disease (CVD) risk when Lp(a) > 30 mg/dL [14]. But there was a contradiction point in LDL-C and Lp(a) control, as statin, the most widely used LDL-C-lowering agent in the world, leading to 15–37% ASCVD risk reduction, could increase Lp(a) by 8.5–19.6% [4, 15]. The clinical benefit of LDL-C lowering with statin could be diminished with the Lp(a) increasing effect. Besides, the optimal clinical control points of LDL-C and Lp(a) is still pending. In this study, we tried to find the relationship between LDL-C, Lp(a) and coronary atherosclerotic lesion in a group of patients with exact evidence of coronary atherosclerosis, and compared correlation strengths within different LDL-C concentrations to find out the balance point of LDL-C and Lp(a) in ASCVD prevention.

## Method

### Study populations 

This was a single-center cross-sectional clinical study of the patients with the diagnosis of coronary atherosclerotic heart disease (CAHD) and performed coronary angiography (CAG) in our medical center. The clinical data and serum samples were collected from patients matching the inclusion and exclusion criteria. Inclusion criteria: (1) Age > 18 years; (2) Conducted CAG between June 2018 and September 2019; (3) Clear awareness, able to sign informed consent and willing to take blood measurements. Exclusion criteria: (1) Previous percutaneous coronary intervention (PCI) or coronary artery bypass graft (CABG); (2) Unstable hemodynamics or left ventricular ejection fraction (LVEF) less than 30%; (3) Severe progressive diseases such as tumors; (4) Rheumatoid or systemic diseases such as sepsis; (5) Cirrhosis or decompensated liver function (Child–Pugh Score > 6); (6) abnormal renal function(estimated glomerular filtration rate < 60 mL/min/1.73m^2^); (7) women of pregnancy and childbearing age. This study is approved by the ethics committee and conducted with signed consent of all participants.

### Study design

**Fig. 1:**
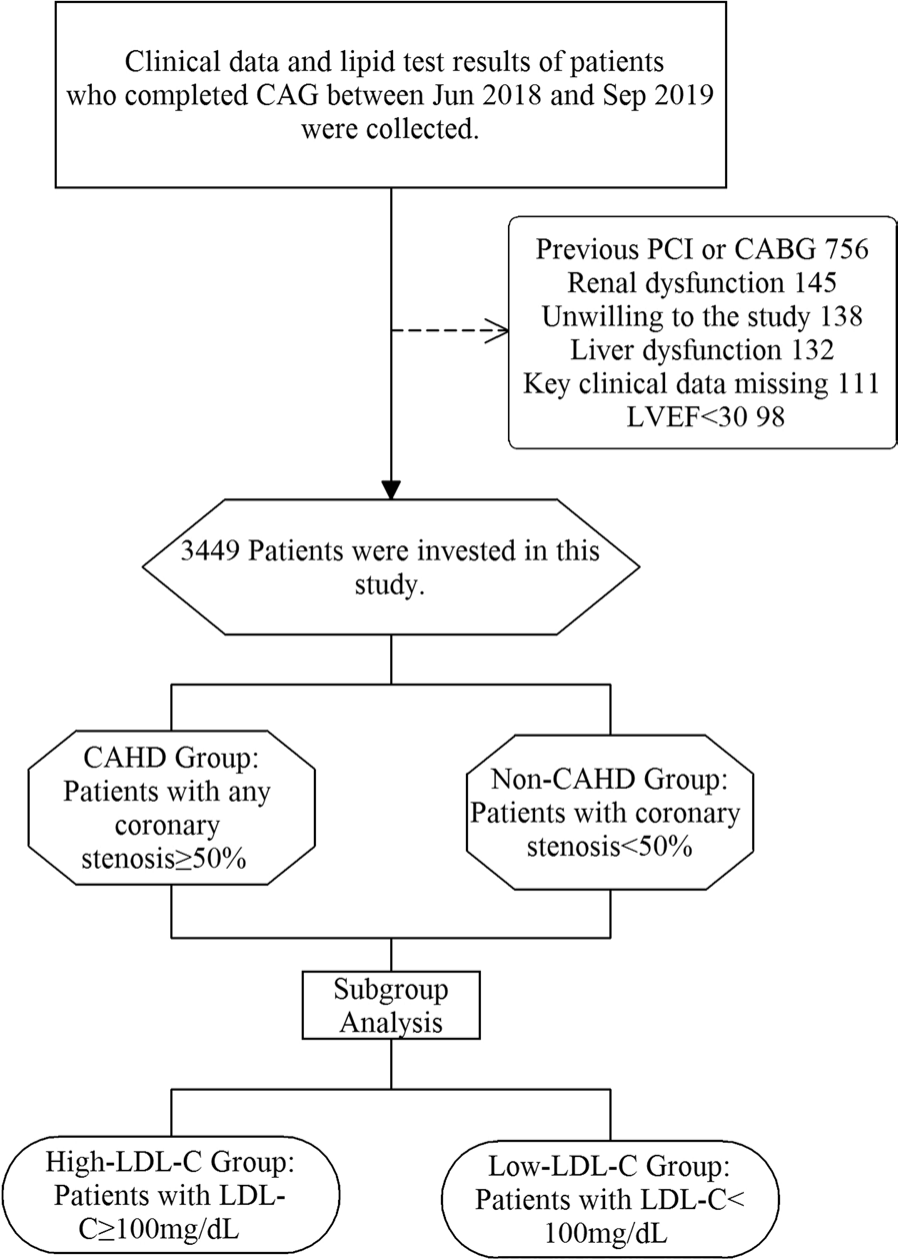
Flow chart. DM, diabetes mellitus; TG, triglyceride; HDL-C, high-density lipoprotein cholesterol; LDL-C, low-density lipoprotein cholesterol; Lp(a), lipoprotein(a); apoA1, apolipoprotein A1; non-HDL-C, total cholesterol minus HDL-C; apoB, apolipoprotein B

Patients with any coronary stenosis ≥ 50% were divided to CAHD Group, with all coronary stenosis < 50% were divided to Non-CAHD Group according to CAG results (Fig. 1). The CAGs were performed for patients who had typical or untypical unstable angina pectoris along with myocardial ischemic changes in electrocardiogram, for example, single or multiple leads/territories ST-segment depression/elevation ≥ 1 mm, increased hyperacute T wave amplitude with prominent symmetrical T waves, pathologic Q waves, cardiac arrhythmias, intraventricular bundle branch blocks, atrioventricular conduction delays, loss of precordial R wave amplitude, etc. Clinical characters and lipid index were compared between the two groups. The clinical characters were collected from medical record system once included in this study and the lipide test results came from clinical standardization laboratory. As most patients (95.3%) are at moderate- or low-risk of CVD according to the 2019 ESC/EAS Guidelines for the management of dyslipidemias [16], we also subdivided patients with the recommended LDL-C concentration and performed subgroup analysis in High-LDL-C Group (LDL-C ≥ 100 mg/dL) and Low-LDL-C Group (LDL-C < 100 mg/dL). Logistic regression analyses were conducted in all patients and subgroups. Spearman correlations were performed to determine the relationship between Lp(a) and Gensini Score. We also conducted Spearman correlation analysis of Lp(a)-Gensini and LDL-C-Gensini in patients with different LDL-C intervals and draw simple dot plots with fitted lines to determine the trends.

### Coronary atherosclerosis lesion and Gensini score

The severity of coronary atherosclerosis lesion was evaluated with Gensini Score [17], which was calculated according to the result of CAG. It was developed to quantitative the severity of the coronary lesions by 3 main parameters: severity score, region multiplying factor and collateral adjustment factor.

### Blood samples collecting and LDL-C, Lp(a) measurement

Fasting venous blood samples were collected before CAG. All samples were centrifugated and stored at − 70 °C. LDL-C was measured in mg/dL by beta quantification. Lp(a) was measured in nmol/L by particle-enhanced turbidimetric immunoassay with Tina-quant Lipoprotein (a) Gen.2 (Latex) (LPA2) Roche® on Cobas system.

### Statistical analysis

Qualitative items were presented as n (%) and quantitative item was presented as mean ± SD. Independent sample t-test was used to determine the difference between quantitative items in different groups and Pearson chi square test was used for the qualitative items. Binary logistic regression was used to evaluate the effects of different variables on CAHD in total patients and subgroup analysis. Before binary logistic regression, whether the logit transformation values of continuous independent variables and dependent variables were linear were test with Box-Tidwell method. Pearson correlation analysis were conducted to determine the relation between Lp(a), LDL-C and Gensini. All statistical analysis was performed with Stata version 15. It was considered statistically significant as *P* < 0.05.

## Results

### Baseline characteristics

3449 patients were invested in this study, of which 678 were distributed to Non-CAHD Group. Except male and diabetes, traditional CAHD risk factors, age, smoking, overweight, hypertension and family history were well matched between two groups (Table 1). Within all patients, 31.8% were divided to High-LDL-C Group. Clinical characteristics comparisons in subgroups was showed in Additional file 1: Tables S1 and S2.

**Table 1:** Clinical characteristics comparisons between non-CAHD and CAHD patients

	Non-CAHD group	CAHD group	*t/χ*^*2*^	*P*
	*N* = 678	*N* = 2771		
Age (y)	63.14 ± 11.51	63.92 ± 11.27	1.609	0.120
Male (No.)	451 (66.52%)	1960 (70.73%)	4.597	0.032
Smoking (No.)	231 (34.07%)	923 (33.31%)	0.142	0.706
Overweight^a^ (No.)	187 (27.58%)	743 (26.81%)	0.163	0.686
Diabetes (No.)	367 (54.13%)	1720 (62.07%)	14.377	< 0.001
Hypertension (No.)	382 (56.34%)	1610 (58.10%)	0.691	0.406
Family history^b^ (No.)	112 (16.52%)	419 (15.12%)	0.818	0.366
TC (mg/dL)	176.27 ± 40.85	175.56 ± 36.31	0.414	0.679
TG (mg/dL)	157.03 ± 81.19	172.20 ± 98.23	4.175	< 0.001
HDL-C (mg/dL)	47.79 ± 8.80	45.08 ± 8.04	7.307	< 0.001
LDL-C (mg/dL)	88.08 ± 33.35	83.94 ± 28.21	2.982	0.003
non-HDL-C (mg/dL)	128.48 ± 38.91	130.48 ± 35.46	1.220	0.223
Lp(a)^c^ (nmol/L)	56.69 ± 62.37	67.88 ± 72.22	4.054	< 0.001
Apo-A1 (mg/dL)	134.04 ± 16.03	128.13 ± 14.97	8.715	< 0.001
Apo-B (mg/dL)	82.44 ± 24.55	84.45 ± 20.81	1.966	0.050

TC, total cholesterol; TG, triglyceride; HDL-C, high-density lipoprotein cholesterol; LDL-C, low-density lipoprotein cholesterol; Lp(a), lipoprotein(a); apo(a)1, apolipoprotein(a)1; apoB, apolipoprotein B

^a^Overweight was defined as body mass index (BMI) > 28 (BMI = weight (kg)/height (m))

^b^Family history was defined as one or more parents or grandparents diagnosed with CAHD

^c^Lp(a) was skewed, and differences between groups are judged by Mann–Whitney U

### Risk FACTORS for CAHD

**Fig. 2:**
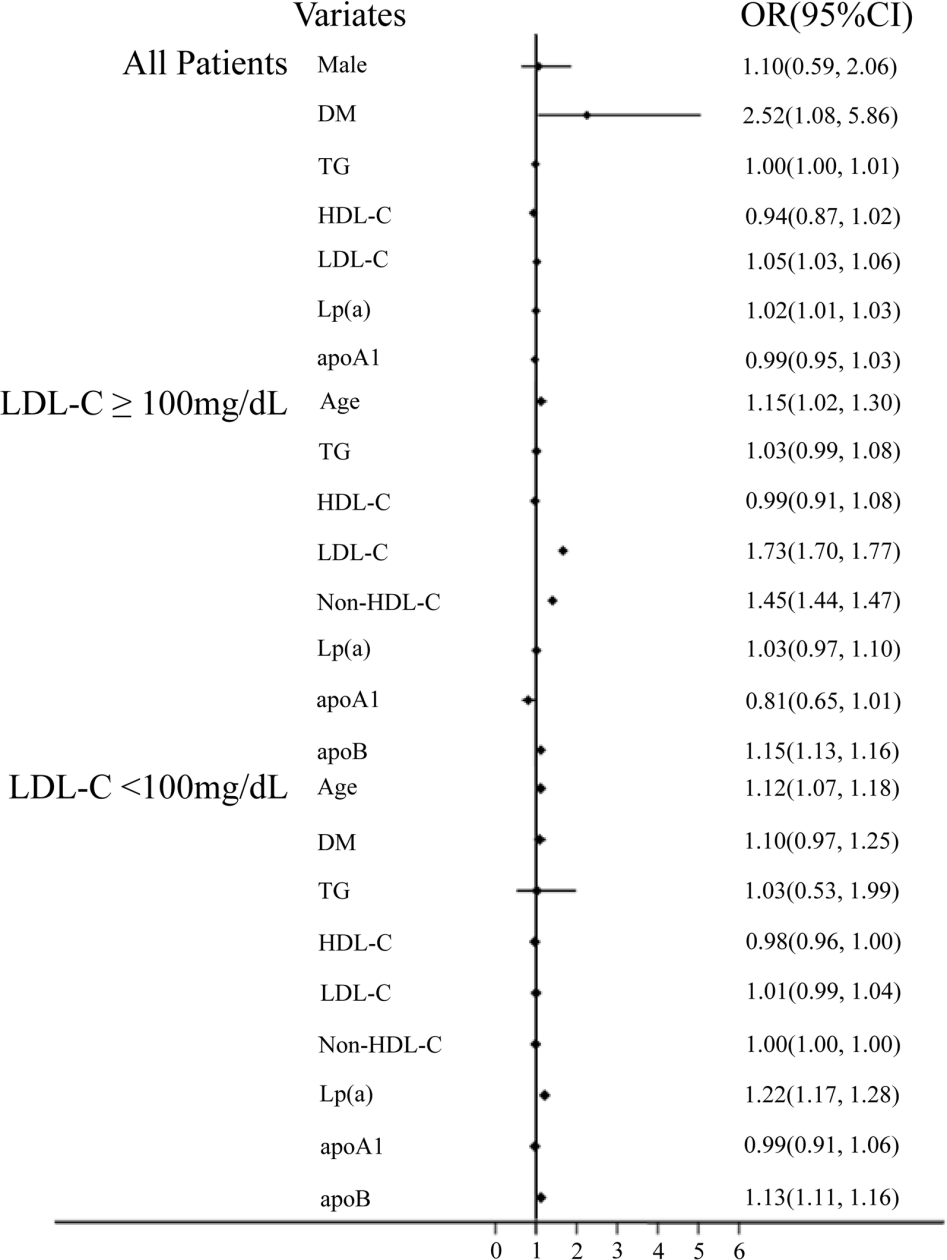
Forest Illustration of Logistic Regression for CAHD in All Patients, High LDL-C Group and Low LDL-C Group. As 95.3% patients are at moderate- or low-risk of CVD, we set LDL-C = 100 mg/dL as the cutoff point as the Guidelines recommended. Low-LDL-C Group: patients with LDL-C < 100 mg/dL. High-LDL-C Group: patients with LDL-C ≥ 100 mg/dL

As showed in Fig. 2, in the Logistic regression of all patients, 3 of 7 variables were risk factors of CAHD, which were diabetes (odds ratio (OR): 2.52, 95% confidence interval (95% CI): 1.08–5.86), LDL-C (OR: 1.05, 95%CI: 1.03–1.06), and Lp(a) (OR: 1.02, 95%CI: 1.01–1.03). In the Logistic regression of High-LDL-C group, 4 of 8 items turned to be risk factors for CAHD, which were age (OR: 1.15, 95%CI: 1.02–1.30), LDL-C (OR: 1.73, 95%CI: 1.70–1.77), non-HDL-C (OR: 1.45, 95%CI: 1.44–1.47), and ApoB (OR: 1.15, 95%CI: 1.13–1.16). While in Low-LDL-C group, among the 9 items, age (OR: 1.12, 95%CI: 1.07–1.18), Lp(a) (OR: 1.22, 95%CI: 1.17–1.28) and ApoB (OR: 1.13, 95%CI: 1.11–1.16) turned out to be CAHD risk factors (Fig. 2).

### Lp(a)-Gensini correlation analysis

**Fig. 3:**
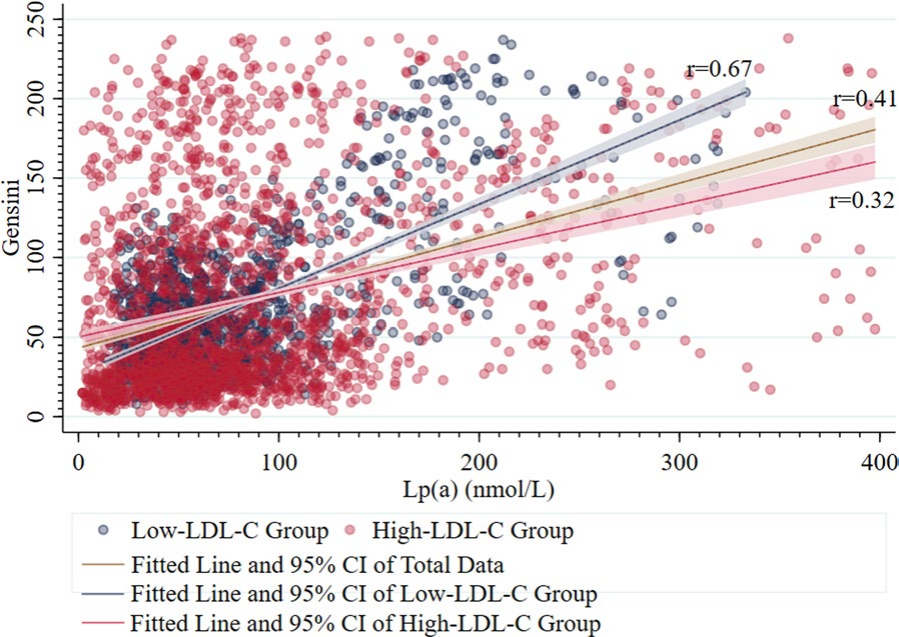
Scatter Plot of Lp(a)-Gensini Correlation in Low-LDL-C Group, High-LDL-C Group and Total Patients. The LDL-C concentration was converted from mg/dL to mmol/L to facilitate the determination of the LDL intervals, with LDL-C (mmol/L) = 0.0259 * LDL-C (mg/dL). r-LDL-C-Gensini: correlation coefficient r in the Spearman correlation analysis of LDL-C and Gensini; r-Lp(a)-Gensini: correlation coefficient r in the Spearman correlation analysis of Lp(a) and Gensini

There were positive linear correlations between Lp(a) and Gensini In Low-LDL-C Group, High-LDL-C Group and total patients with the Spearman correlation coefficient r of 0.67, 0.32 and 0.41, (*P* < 0.001). Lp(a) was more relevant to Gensini in Low-LDL-C Group than the other two group (*P* < 0.001), but there was no significant difference between total patients and High-LDL-C Group (*P* = 0.07) (Fig. 3).

### Lp(a)-Gensini and LDL-C-Gensini correlation in different LDL-C intervals

**Fig. 4:**
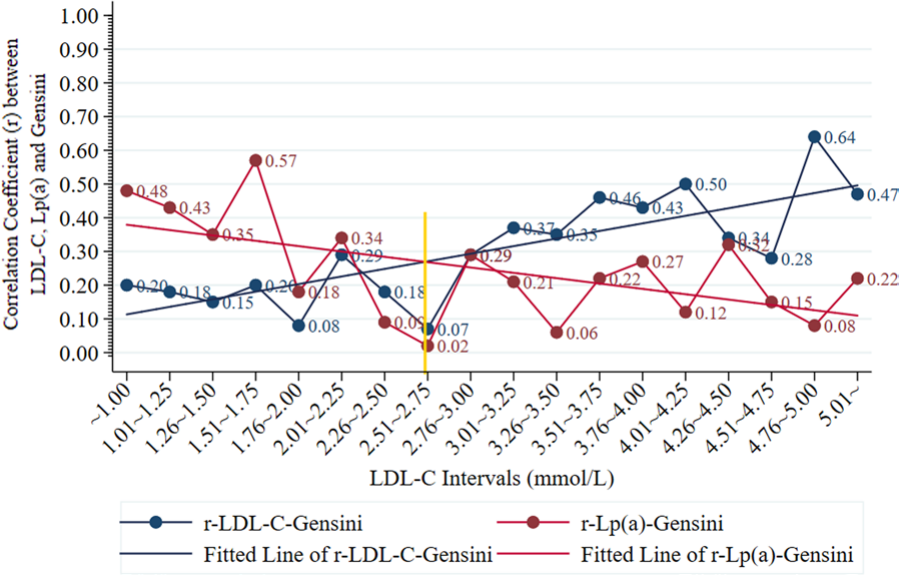
Simple Scatter plot with Fitted Lines of the Correlation Coefficient rs in the Pearson Correlation of LDL-C, Lp(a) and Gensini in different LDL-C Intervals

The LDL-C-Gensini correlation increased along with LDL-C concentration increase. While the correlation of Lp(a)-Gensini decreased with increasing LDL-C level (Fig. 4). The highest coefficient r of LDL-C-Gensini correlation was 0.64 in the LDL-C of 4.76–5.00 mmol/L, while the lowest r was 0.07 within the LDL-C of 2.51–2.75 mmol/L. The coefficient r of Lp(a)-Gensini correlation ranged in 0.02–0.57, with r = 0.02 in the LDL-C concentration of 2.51–2.75 mmol/L and r = 0.57 in 1.51–1.75 mmol/L. The fitted lines of LDL-C-Gensini and Lp(a)-Gensini correlation coefficient rs crossed near the point of LDL-C = 2.7 mmol/L. The LDL-C intervals were set as 0.25 mmol/L to meet the minimum request for Spearman correlation analysis, and the number of patients in different LDL-C intervals ranked from 35, in both 4.51–4.75 mmol/L and 5.01 mmol/L intervals, to 450 in 3.26–3.50 mmol/L.

## Discussion

In this study, we found the distinct CAHD risk factors in patients with different LDL-C levels. Moreover, in the correlation analysis, we found the linear correlation between Lp(a), LDL-C and Gensini and the correlation was influenced by LDL-C concentration. A meta-analysis of 49 clinical trials, over 312,000 patients, showed a great 23% MACE risk reduction achievement with 1 mmol/L LDL-C reduction in statin therapy [18]. But under statin therapy background, additional LDL-C lowering with evolocumab, a proprotein convertase subtilisin/kexin type 9 (PCSK9) inhibitor, achieved additional 60% LDL-C reduction but only 1.5% MACE risk reduction [5]. Those results indicated that the clinical benefit of LDL-C lowering therapy descended with LDL-C decrease which is consistent with our finding in this study that the correlation between LDL-C and Gensini dropped with the LDL-C level decrease.

Even the Guideline recommended LDL-C concentration was strict for patients at very-high CVD risk, < 55 mg/dl, in both primary and secondary prevention [16]. The data in the secondary prevention of vascular disease showed that the recurrent 10-year risk of vascular events is still over 30% in 9% patients with vascular disease, who’s risk factors were all at guideline-recommended targets [19]. Lp(a) may contribute to the residual risk. In a recently published epidemiological study, Hu etc. fund the Lp(a) co-contributed with LDL-C to the incidence of acute myocardial infarction in Chinese people [20]. In this study, we found the same trend, especially in patients with LDL-C < 100 mg/dL. In the subgroup analysis of Low-LDL-C Group in this study, near 43% patients were taking statin, the pathogenicity of Lp(a) may partly due to the effect of statins on Lp(a) increasing.

Lp(a) is mainly composed with an apolipoprotein a (apo(a)), an LDL like particle and phospholipid (PL). Under the stimulation of inflammation, LDL and PL enter the vascular endothelium and convert to oxidized LDL (Ox-LDL) and oxidized phospholipids (Ox-PL), which are critical in the process of atherosclerosis. Lp(a) can induce and accelerate atherothrombosis beyond its LDL components and is more effective than LDL in atherosclerosis inducing. However, 70–80% patients with the risk of CVD have low Lp(a) level and LDL-C present in significant excess to Lp(a), the LDL-driven CVD risk is mainly due to LDL-C. But the traditional clinical panel couldn’t distinguish Lp(a) from LDL-C, 30–45% of the reported LDL-C is contributed by Lp(a). In more extreme cases, the majority of LDL-C was carried by Lp(a) when LDL-C less than 25 mg/dL[21]. In our study, Lp(a) showed no significant relation to CAHD in High-LDL-C Group but a strong relation to CAHD in patients with LDL-C < 100 mg/dL. That was partly because the weakened pathogenicity of LDL-C in the Low-LDL-C Group and the particle-enhanced turbidimetric immunoassay measurement of Lp(a), we adopted in this study.

The apo(a) component of Lp(a) contains three kringles, KII, KIV and KV. Among the 10 subtypes of KIV, KIV 1–10, there are large variations in the copies of KIV 2 domain, leading to over 40 different sizes of Lp(a). It was reported that over 80% individuals caring 2 different-size apo (a) isoforms [22]. The common clinical report of Lp(a) was in total mass (mg/dL), but it has significant limitation of bias in measurement, considering the variable of Lp(a) components among patients. Thus, the National Heart, Lung, and Blood Institute (NHLBI) Working group recommended that Lp(a) should be reported in particle concentration [23]. The measurement of Lp(a) in this study was based on a latex coated antibody of lipoprotein, Tina-quant Lipoprotein (a) Gen.2, which is free from the influence of Lpa(a) polymorphism, and the accuracy is higher among six common commercial measurements [24].

A prospective study suggested that the relationship between Lp(a) and CVD was a J-curve with a low slop when Lp(a) level was very low and sharply raised when Lp(a) level increased [25]. Dr. Tsimikas thought the correlation of CVD risk and circulating Lp(a) mass is in a linear ship, when Lp(a) level increased over 25 mg/dl [26]. As the result showed in this study, the molar concentration of Lp(a) correlated with coronary atherosclerotic lesions in a linear shape, but it was influenced by LDL-C level. We also noticed that both the correlations of Lp(a)-Gensini, and LDL-C-Gensini were weakest in LDL-C = 2.51–2.75 mmol/L, which is near to the recommended LDL-C level for patients with low CVD risk [16]. The reason for that phenomenon may be the advantage number of non-statins intervened Non-CAHD patients in this subgroup.

In this study, we set the 50% coronary stenosis, measured in naked eye, as the as grouping basis. There would be errors in the grouping of patients with borderline lesions, without the intravascular imaging examination. 37% of the total patients in this study were under statin therapy and the duration of medication varied, the effect of statin on Lp(a) cannot be determined. In the correlation analysis of Lp(a)- Gensini and LDL-C-Gensini in different LDL-C intervals, the numbers of patients varied greatly among subgroups, leaded to unavoidable measurement bias. Besides, our study just proved the strong correlation between Lp(a) and CAHD in low LDL-C patients, but hardly confirm the clinical benefits of Lp(a) lowering intervention in those patients.

## Conclusion

In this study, we found the Lp(a) was the risk factor of coronary atherosclerosis heart disease in patients with LDL-C < 100 mg/dL, rather than in patients with LDL-C ≥ 100 mg/dL. The correlation between Lp(a) and coronary atherosclerosis lesion was influenced by LDL-C concentration, and the correlation was stronger than LDL-C when LDL-C less than 104 mg/dl. In the patients with LDL-C < 104 mg/dL, whether the Lp(a) based intervention can achieve clinical benefits remains to be further studied.

## Supplementary Information

**Additional file 1.** **Supplement Table 1.** Baseline Characteristics Comparisons of Patients with LDL-C ≥ 100 mg/dL in Non-CAHD Group and CAHD Group. **Supplement Table 2.** Baseline Characteristics Comparisons of Patients with LDL-C < 100 mg/dL in Non-CAHD Group and CAHD Group.

## Data Availability

All data generated or analyzed during this study are included in this published article.

## Abbreviations

ASCVD: Atherosclerotic cardiovascular disease
LDL-C: Low-density lipoprotein cholesterol
MACE: Major cardiovascular adverse event
Lp(a): Lipoprotein(a)
CVD: Cardiovascular disease
CAHD: Coronary atherosclerotic heart disease
CAG: Coronary angiography
PCSK9: Proprotein convertase subtilisin/kexin type 9
apo(a): Apolipoprotein(a)
