# Supplementary material for: The correlation between lipoprotein(a) and coronary atherosclerotic lesion is stronger than LDL-C, when LDL-C is less than 104 mg/dL
Source: BMC Cardiovasc Disord. 2021 Jan 19;21:41. doi: 10.1186/s12872-021-01861-6 (PMC7816444; doi:10.1186/s12872-021-01861-6)
Supplement: Supplementary file 1 — Additional file 1. Supplement Table 1. Baseline Characteristics Comparisons of Patients with LDL-C ≥ 100 mg/dL in Non-CAHD Group and CAHD Group. Supplement Table 2. Baseline Characteristics Comparisons of Patients with LDL-C < 100 mg/dL in Non-CAHD Group and CAHD Group. [file 12872_2021_1861_MOESM1_ESM.docx]

Supplement Table 1 Baseline Characteristics Comparisons of Patients with LDL-C≥100mg/dL in Non-CAHD Group and CAHD Group

|  | Non-CAHD Group | CAHD Group | *t/χ^2^* | *P* |
| --- | --- | --- | --- | --- |
|  | *N* = 250 | *N* = 847 |  |  |
| Age, y | 64.12 ± 7.96 | 65.38 ± 8.59 | 2.071 | 0.039 |
| Male, No. | 163(65.20%) | 605(71.43%) | 3.567 | 0.059 |
| Smoking, No. | 83(33.20%) | 285(33.65%) | 0.017 | 0.895 |
| Overweight ^a^, No. | 70(28.00%) | 233(27.51%) | 0.023 | 0.879 |
| Diabetes, No. | 134(53.60%) | 510(60.21%) | 3.482 | 0.062 |
| Hypertension, No. | 138(55.20%) | 500(59.03%) | 1.165 | 0.280 |
| Family history ^b^, No. | 46(18.40%) | 149(17.59%) | 0.086 | 0.769 |
| TC(mg/dL) | 208.66 ± 22.32 | 209.71 ± 28.43 | 0.612 | 0.541 |
| TG(mg/dL) | 162.55 ± 62.64 | 187.85 ± 84.83 | 5.144 | <0.001 |
| HDL-C(mg/dL) | 52.10 ± 8.78 | 50.86 ± 8.57 | 1.999 | 0.046 |
| LDL-C(mg/dL) | 115.57 ± 9.44 | 120.16 ± 17.92 | 5.352 | <0.001 |
| non-HDL-C(mg/dL) | 149.56 ± 19.92 | 159.83 ± 28.63 | 6.425 | <0.001 |
| Lp(a) ^c^(nmol/L) | 53.50 ± 59.10 | 61.10 ± 79.37 | 12.449 | <0.001 |
| Apo-A1(mg/dL) | 140.37 ± 17.45 | 135.13 ± 16.08 | 4.439 | <0.001 |
| apoB (mg/dL) | 94.23 ± 13.41 | 102.18 ± 19.18 | 7.402 | <0.001 |

a, Overweight was defined as body mass index (BMI) > 28 (BMI = weight (Kg) / height (m))

b, Family history was defined as one or more parents or grandparents diagnosed with CAHD;

c, Lp(a) was skewed, and differences between groups are judged by Mann-Whitney U.

TC, total cholesterol; TG, triglyceride; HDL-C, high-density lipoprotein cholesterol; LDL-C, low-density lipoprotein cholesterol; Lp(a), lipoprotein(a); apo(a)1, apolipoprotein(a)1; apoB, apolipoprotein B

Supplement Table 2 Baseline Characteristics Comparisons of Patients with LDL-C<100mg/dL in Non-CAHD Group and CAHD Group

|  | Non-CAHD Group | CAHD Group | *t/χ^2^* | *P* |
| --- | --- | --- | --- | --- |
|  | *N* = 428 | *N* = 1924 |  |  |
| Age, y | 62.50 ± 8.03 | 63.75 ± 4.55 | 3.111 | 0.002 |
| Male, No. | 288(67.29%) | 1355(70.43%) | 1.636 | 0.201 |
| Smoking, No. | 148(34.58%) | 638(33.60%) | 0.317 | 0.573 |
| Overweight ^a^, No. | 117(27.34%) | 510(26.51%) | 0.123 | 0.726 |
| Diabetes, No. | 233(54.44%) | 1210(62.89%) | 10.545 | 0.001 |
| Hypertension, No. | 244(57.01%) | 1110(57.69%) | 0.067 | 0.796 |
| Family history ^b^, No. | 66(15.42%) | 270(14.03%) | 0.550 | 0.458 |
| TC(mg/dL) | 141.10 ± 24.61 | 148.68 ± 59.49 | 4.202 | <0.001 |
| TG(mg/dL) | 143.92 ± 68.99 | 148.87 ± 57.34 | 1.382 | 0.168 |
| HDL-C(mg/dL) | 44.24 ± 8.56 | 50.22 ± 6.96 | 13.494 | <0.001 |
| LDL-C(mg/dL) | 65.57 ± 20.08 | 74.98 ± 19.70 | 2.449 | 0.015 |
| non-HDL-C(mg/dL) | 103.09 ± 24.43 | 113.46 ± 52.63 | 6.160 | <0.001 |
| Lp(a) ^c^(nmol/L) | 61.40 ± 65.31 | 72.40 ± 63.57 | 3.222 | 0.001 |
| Apo-A1(mg/dL) | 130.94 ± 13.47 | 125.25 ± 15.56 | 7.674 | <0.001 |
| apoB (mg/dL) | 65.34 ± 14.42 | 69.52 ± 23.81 | 4.731 | <0.001 |

a, Overweight was defined as body mass index (BMI) > 28 (BMI = weight (Kg) / height (m))

b, Family history was defined as one or more parents or grandparents diagnosed with CAHD;

c, Lp(a) was skewed, and differences between groups are judged by Mann-Whitney U.

TC, total cholesterol; TG, triglyceride; HDL-C, high-density lipoprotein cholesterol; LDL-C, low-density lipoprotein cholesterol; Lp(a), lipoprotein(a); apo(a)1, apolipoprotein(a)1; apoB, apolipoprotein B
